# Pre-hypertension and Associated Risk Factors Among Undergraduate Medical Students in Chengalpattu District: A Cross-Sectional Study

**DOI:** 10.7759/cureus.85896

**Published:** 2025-06-13

**Authors:** Rehana Syed, Raja D, Manoj P, BN Surya, Hari Narayanan, Geethanjali Murthy

**Affiliations:** 1 Epidemiology and Public Health, Chettinad Hospital and Research Institute, Chettinad Academy of Research and Education, Kelambakkam, IND

**Keywords:** adolescent health, elevated hypertension, non-communicable disease, perceived stress, systemic hypertension, young adults

## Abstract

Introduction

Noncommunicable diseases (NCDs) are a major global health concern, with cardiovascular conditions being a leading contributor. Medical students are exposed to greater stress in terms of longer course duration as well as academic stress. Medical professionals adopt unhealthy lifestyles and end up developing chronic diseases at a younger age. This study screens such a vulnerable population for hypertension and aids in early diagnosis. Hypertension plays a key role in this burden, and its early stage (pre-hypertension) is recognized as a significant risk factor for developing full-blown hypertension and related complications. This study focuses on assessing the prevalence of pre-hypertension and identifying its associated factors among undergraduate medical students in South India.

Aim

To assess the prevalence of pre-hypertension among undergraduate medical college students in Chengalpattu district, Tamil Nadu, India. This study also aims to find out the various risk factors associated with pre-hypertension among undergraduate medical college students in Chengalpattu district.

Methodology

A cross-sectional study was conducted among 350 students from a medical college in Chengalpattu district. Participants were selected randomly, and data were collected using a pretested and structured questionnaire.

Results

Among the 350 study participants, 28% had pre-hypertension, 67.7% were normotensive, and 4.3% had stage 1 hypertension and were excluded from treatment. Pre-hypertension was significantly associated with age above 22 (AOR =1.833), in the final year (AOR = 2.107), smoking (AOR = 2.755), stress (AOR = 3.822), poor sleep (AOR = 1.983), inactivity (AOR = 2.738), and family history of hypertension (AOR = 2.186). Stress (AOR = 3.822) and smoking were the strongest predictors (AOR = 2.755). Stress and smoking were the strongest predictors but could not be quantified. Researchers could further explore this and create awareness. The study highlights the need for early intervention in high-risk groups.

Conclusion

The high prevalence of pre-hypertension and its strong links to both modifiable and demographic risk factors among medical students highlight the need for timely and focused preventive measures. The medical curriculum could adopt screening health campaigns and life skill education workshops. Early screening, lifestyle interventions, and stress management initiatives within academic settings are crucial in mitigating long-term cardiovascular risks and ensuring the health of future healthcare professionals.

## Introduction

Globally, non-communicable diseases (NCDs) are the main contributors to morbidity and mortality. According to a World Health Organization (WHO) fact sheet, NCDs cause 40 million deaths a year, or 70% of all fatalities worldwide [1], with over 80% of those deaths taking place in low- and middle-income nations.

The leading cause of mortality worldwide among the NCDs is cardiovascular disease (CVD) [2]. The World Health Organization defines hypertension (HTN) as a cardiovascular illness in which the blood vessels are under greater stress due to consistently elevated pressure [3]. AT least 9.4 million deaths worldwide are attributed to complications from hypertension each year [4].

In India, the burden is substantial: a multicity survey across five major regions reported prehypertension rates of approximately 31-35% in South and West India, with Mumbai and Trivandrum showing similar elevations compared to northern regions [5]. South India, particularly urban areas, demonstrates both high prevalence and low awareness: a STEPS (STEPwise approach to NCD risk factor surveillance) community survey in Puducherry reported a 36.6% prehypertension rate, significantly associated with BMI > 23 kg/m² and daily salt intake over 6 g [6]. Cross-sectional data from coastal Karnataka showed nearly 55% of adults in the prehypertensive or hypertensive range, related to sedentary habits, obesity, alcohol, and tobacco use [7]. Among undergraduate medical students (a young but high‑stress group) the prevalence is often higher than in peers, with studies reporting 38% (Puducherry) to 37.5% (Davangere), linked to BMI, dyslipidemia, and stress [8,9].

The American term "pre-hypertension" refers to a condition in which a person's blood pressure is higher than normal (systolic blood pressure of 120-139 mmHg or diastolic blood pressure of 80-89 mmHg) but not high enough to be classified as hypertension as per JNC criteria 7 (Seventh Joint National Committee) [10]. According to several research carried out in India, the prevalence of pre-HTN in people aged 20 to 30 ranges from 24.6% to 65% [11-13]. There is a moderate to high chance that pre-hypertensives will develop into hypertension, and a significant percentage of them have at least one cardiovascular risk factor [11-13].

Early identification of pre-hypertension plays an important role in screening for metabolic syndrome and prevention of cardiovascular accident [14-16]. Independent of other cardiovascular risk factors, pre-hypertension increases the chance of major cardiovascular events and the likelihood of developing hypertension later in life. The purpose of this study is to determine the prevalence of prehypertension and related variables among South Indian undergraduate medical students.

## Materials and methods

Study design, setting, and duration

This cross-sectional study was conducted from February 2024 to May 2024 among 350 undergraduate students from two colleges in the Chengalpattu district, Tamil Nadu, India.

Sample size calculation

A sample size of 350 was determined based on a previous study by Shetty and Nayak, which reported a 55.4% prevalence of pre-hypertension [17]. The sample size calculation considered a relative precision of 10%, with an additional 10% included for non-responsiveness.

Sampling procedure

Among eight blocks in Chengalpattu district, four blocks were randomly selected by lottery method using simple random sampling. A list of medical colleges in each block was obtained from the college database. Two colleges were selected randomly by the coin toss method. In the Indian medical education system, the undergraduate program typically follows a structured curriculum regulated by national councils. The Bachelor of Medicine and Bachelor of Surgery (MBBS) program spans 5.5 years, including 4.5 years of academic courses followed by a one-year compulsory rotating internship. The sampling frame included all students from the selected colleges. Three hundred and fifty students were randomly chosen from the sampling frame using a simple random sampling method.

**Figure 1:**
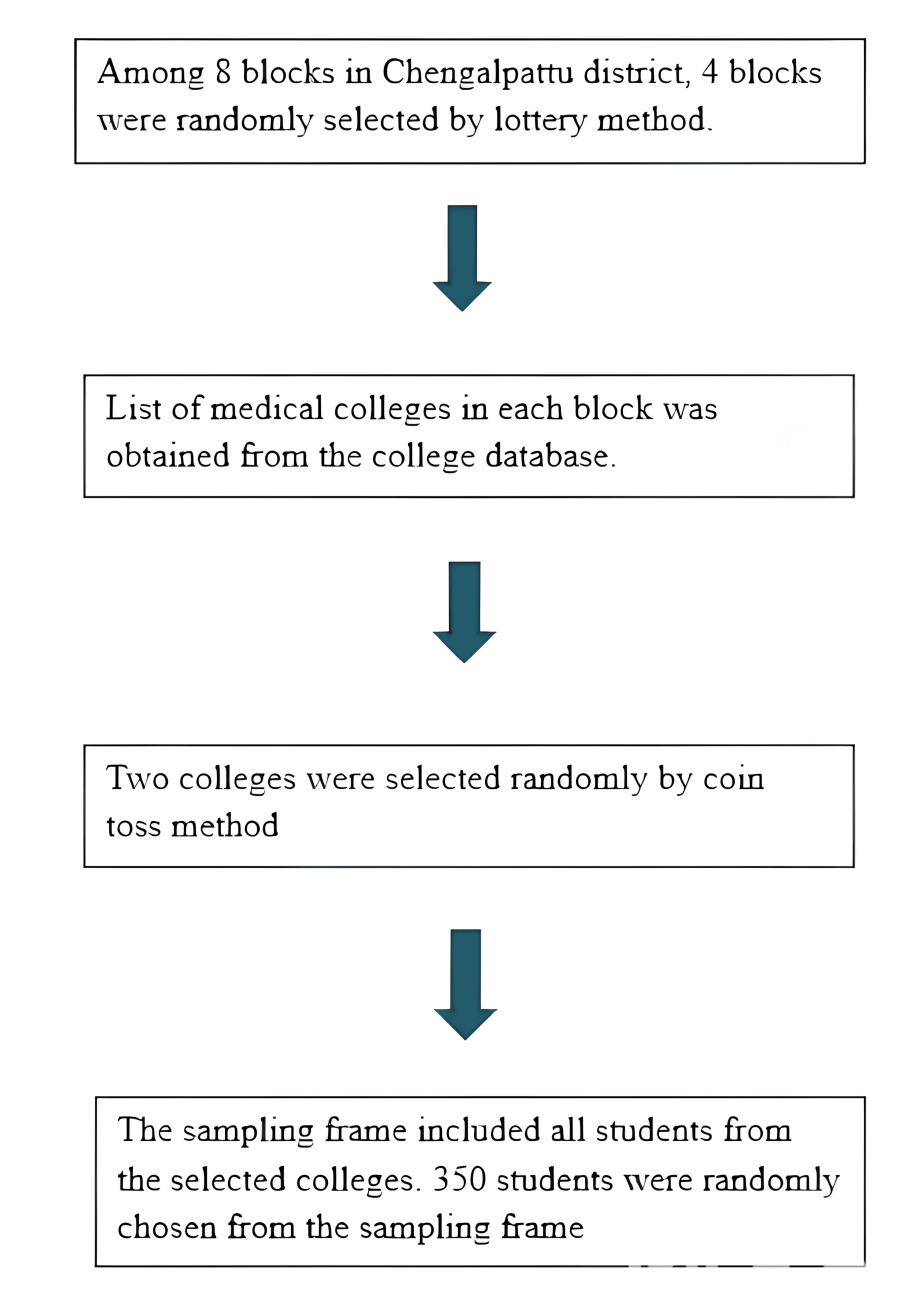
Flowchart of sampling procedure

Inclusion and exclusion criteria**

First-year to final-year MBBS students above 18 years of age were included, while those with a known history of hypertension under medication were excluded from the study.

Data collection

Permission to conduct the study was formally obtained from the heads of the selected institutions. The objectives of the study were explained to the administrative authorities and students, and informed consent was obtained from all participants before data collection. Data was collected using a pre-tested semi-structured questionnaire (see Appendices) covering (A) socio-demographic details, (B) anthropometric details, (C) determinants of pre-hypertension (physical activity, hours of sleep, BMI was measured using the Quetelet Index, and (D) 10-item Perceived Stress Scale (PSS-10) [18]. BP was measured using a standard digital sphygmomanometer which was calibrated using stride BP every 15 days once (Omron HBP 1320, Omron Healthcare India Pvt. Ltd, Gurgaon, India), with participants seated and at rest. Each participant’s blood pressure was recorded twice, and the average was taken for analysis. The JNC 7 (Seventh Joint National Committee) guidelines were used to classify blood pressure levels [10].

A pilot study was conducted among 30 participants using a semi-structured questionnaire among college students from a different university. Based on the responses and feedback received the questionnaire was modified. The internal validity of the questionnaire was analyzed using reliability analysis. The Cronbach’s alpha of the questionnaire developed was 0.82, which indicated good consistency in internal validity.

To assess levels of perceived stress among the study participants, PSS developed by Cohen et al. [18]** **was used. The PSS 10-item scale includes questions about participants’ stressful feelings and thoughts over the past month, and yields scores that reflect one's perceived stress. Reverse scoring is applied to questions 4, 5, 7, and 8, adjusting the scale to range from 0 to 4. The total score, ranging from 0 to 40, is then computed by summing individual item scores. Higher PSS scores correlate with heightened perceived stress, where a score of 0-13 is considered low stress, 14-26 indicates moderate stress, and 27-40 suggests high perceived stress. The 10‑item PSS version was chosen due to its notable good psychometric properties and the evidence of its validity [19].

Ethical consideration and statistical analysis

Ethical approval from the institutional human ethical committee was obtained (Ref no: IHEC-I/2491/24) before commencing data collection. Informed written consent was secured from all participants. collected data was analyzed using IBM SPSS Statistics for Windows, Version 27 (IBM Inc., Armonk, NY, USA). Bivariate logistic regression was performed to obtain an unadjusted odds ratio. Variables with a p-value <0.05 were included in the multivariate model to calculate the adjusted odds ratio.

## Results

Table 1 shows the distribution details of the study participants, out of 350 participants, 48% were male individuals and 52% were female individuals. Most (64%) were aged 22 years or younger. Regarding their year of study, 29.7% were in the fourth year, followed by 27.7% in the second year, 22.6% in the third year, and 20% in the first year. A majority (58.3%) belonged to nuclear families, while 41.7% were from joint families. A history of alcohol consumption was reported by 18% of participants and smoking by 13.4%. In terms of residence, 42.9% were hostellers and 57.1% were day scholars.

**Table 1:** Socio-demographic variables of the study participants

S. No.	Variable	Frequency (n=350)	Percentage (%)
1.	Gender		
	Male	168	48 (%)
	Female	182	52 (%)
2.	Age		
	≤ 22 years	224	64 (%)
	> 22 years	126	36 (%)
3.	Year of study		
	First year	70	20 (%)
	Second year	97	27.7 (%)
	Third year	79	22.6 (%)
	Final year	104	29.7 (%)
4.	Type of family		
	Nuclear	204	58.3 (%)
	Joint	146	41.7 (%)
5.	History of alcohol consumption		
	Present	63	18 (%)
	Absent	287	82 (%)
6.	History of smoking		
	Present	47	13.4 (%)
	Absent	303	86.6 (%)
7.	Type of stay		
	Hosteller	150	42.9 (%)
	Day scholar	200	57.1 (%)

**Figure 2:**
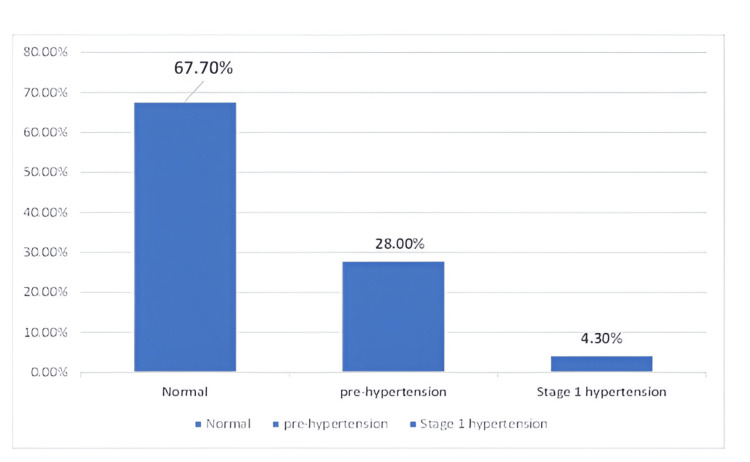
Grades of hypertension

Figure 2 shows the prevalence of pre-hypertension. It was found that 98 (28%) of 350 study participants had pre-hypertension, while 67.7% (237) and 4.3% (15) had stage 1 hypertension. The 15 participants identified with stage 1 hypertension were excluded from further analysis, and appropriate treatment and counseling were initiated for them.

Table 2 shows the association between pre-hypertension and various demographic variables among the study participants. Individuals aged above 22 years had higher odds of pre-hypertension compared to those aged 22 years or below (odds ratio = 1.769, 95% CI: 1.09-2.86, p = 0.019). Female participants demonstrated a significantly higher likelihood of pre-hypertension compared to male participants (odds ratio = 1.767, 95% CI: 1.08-2.86, p = 0.020). Fourth-year students were more likely to have pre-hypertension compared to first-year students (odds ratio = 2.171, 95% CI: 1.08-4.37, p = 0.030). Smoking habits were significantly associated with pre-hypertension, with smokers having higher odds compared to non-smokers (odds ratio = 2.492, 95% CI: 1.38-4.49, p = 0.002).

**Table 2:** Association between pre-hypertension and socio-demographic details * P value < 0.05, statistically significant at 95% confidence interval. OR: odds ratio, χ2: Chi-square

S. No.	Variable	Pre-hypertension n (%) n = 98 (29.3%)	Normal n (%) n = 237 (70.7%)	Total (N = 335)	Chi-square	OR (95% CI)	P value
1.	Age						
	> 22 years	46 (46.9%)	79 (33.3%)	125 (37.3%)	5.487	1.769 (1.09 – 2.86)	0.019*
	≤ 22 years	52 (53.1%)	158 (66.7%)	210 (62.7%)		1	1
2.	Gender						
	Female	62 (63.3%)	117 (49.4%)	179 (53.4%)	5.382	1.767 (1.08 – 2.86)	0.020*
	Male	36 (36.7%)	120 (50.6%)	156 (46.6%)		1	1
3.	Year of study						
	Fourth year	38 (38.8%)	63 (26.6%)	101 (30.1%)	8.174	2.171 (1.08 – 4.37)	0.030*
	Third year	15 (15.3%)	57 (24.1%)	72 (21.5%)		0.947 (0.42 – 2.12)	0.895
	Second year	30 (30.6%)	63 (26.6%)	93 (27.8%)		1.741 (0.84 – 3.52)	0.141
	First year	15 (15.3%)	54 (22.8%)	69 (20.6%)		1	1
4.	Type of family						
	Nuclear	53 (54.1%)	144 (60.8%)	197 (58.8%)	1.276	0.761 (0.47 – 1.22)	0.259
	Joint	45 (45.9%)	93 (39.2%)	138 (41.2%)		1	1
5.	Smoking habit						
	Yes	26 (26.5%)	30 (12.7%)	56 (16.7%)	9.584	2.492 (1.38 – 4.49)	0.002*
	No	72 (73.5%)	207 (87.3%)	279 (83.3%)		1	1
6.	Alcohol consumption						
	Yes	18 (18.4%)	28 (11.8%)	46 (13.7%)	2.513	1.679 (0.88 – 3.20)	0.113
	No	80 (81.6%)	209 (88.2%)	289 (86.3%)		1	1
7.	Type of stay						
	Day scholar	58 (59.2%)	131 (55.3%)	189 (56.4%)	0.431	1.173 (0.73 – 1.89)	0.512
	Hosteller	40 (40.8%)	106 (44.7%)	146 (43.6%)		1	1

Table 3 highlights significant associations between pre-hypertension and various factors among the study participants. A family history of hypertension was significantly associated with higher odds of pre-hypertension (OR = 1.809, 95% CI: 1.12-2.93, p = 0.015). Participants engaging in physical activity less frequently, such as never or once a week, were at greater risk compared to those exercising more than five times a week (OR = 4.345, p = 0.032; OR = 3.672, p = 0.047, respectively). Sleeping less than six hours per day had a positive association with pre-hypertension (OR = 1.820, 95% CI: 1.08-3.07, p = 0.024). Additionally, stress (measured by the PSS-10 scale) was strongly linked to pre-hypertension, with participants experiencing stress having more than three times higher odds (OR = 2.020, 95% CI: 1.23 - 3.21, p 0.005).

**Table 3:** Association between pre-hypertension and lifestyle factors * P value < 0.05, statistically significant at 95% confidence interval. OR: odds ratio, χ2: Chi-square

S. No.	Variable	Pre-hypertension n (%) n = 98 (29.3%)	Normal n (%) n = 237 (70.7%)		Total (N = 335)	Chi-square	OR (95% CI)	P value
1.	Family history of hypertension							
	Yes	61 (62.2%)		113 (47.7%)	174 (51.9%)	5.892	1.809 (1.12 – 2.93)	0.015*
	No	37 (37.8%)		124 (52.3%)	161 (48.1%)		1	1
2.	BMI							
	Obese	11 (11.2%)		18 (7.6%)	29 (8.7%)	1.944	1.390 (0.61 – 3.15)	0.431
	Overweight	36 (36.7%)		103 (43.5%)	139 (41.5%)		0.795 (0.48 – 1.31)	0.371
	Normal	51(52%)		116 (48.9%)	167 (49.9%)		1	1
3.	Physical activity/week							
	Never	21 (21.4%)		29 (12.2%)	50 (14.9%)	16.196	4.345 (1.13 – 16.68)	0.032*
	Once a week	41 (41.8%)		67 (28.3%)	108 (32.2%)		3.672 (1.02 – 13.24)	0.047*
	Twice a week	25 (25.5%)		80 (33.8%)	105 (31.3%)		1.875 (0.51 – 6.89)	0.344
	3-4 times a week	8 (8.2%)		43 (18.1%)	51 (15.2%)		1.116 (0.26 – 4.69)	0.881
	> 5 times a week	3 (3.1%)		18 (7.6%)	21 (6.3%)		1	1
4.	Hours of sleep/day							
	< 6 hours	73 (74.5%)		146 (61.6%)	219 (65.4%)	5.086	1.820 (1.08 – 3.07)	0.024*
	> 6 hours	25 (25.5%)		91 (38.4%)	116 (34.6%)		1	1
5.	Type of diet							
	Mixed	72 (73.5%)		183 (77.2%)	255 (76.1%)	0.535	0.817 (0.48 – 1.40)	0.464
	Vegetarian	26 (26.5%)		54 (22.8%)	80 (23.9%)		1	1
6.	How often you consume junk foods/week							
	> 5 times	17 (17.3%)		24 (10.1%)	41 (12.2%)	3.560	1.574 (0.58 – 4.29)	0.375
	3-4 times	41 (41.8%)		111 (46.8%)	152 (45.4%)		0.82 (0.35 – 1.95)	0.654
	1-2 times	31 (31.6%)		82 (34.6%)	113 (33.7%)		0.840 (0.35 – 2.04)	0.701
	Never	9 (9.2%)		20 (8.4%)	29 (8.7%)		1	1
7.	Stress (PSS – 10 scale)							
	Yes	65 (66.3%)		117 (49.4%)	182 (54.3%)	8.037	2.020 (1.23 – 3.29)	0.005*
	No	33 (33.7%)		120 (50.6%)	153 (46.7%)		1	1

Table 4 presents the adjusted analysis of factors associated with pre-hypertension among study participants. Participants older than 22 years had significantly higher odds of pre-hypertension (AOR 1.83; 95 % CI 1.03-2.60; p = 0.034). Final‑year MBBS students were also at increased risk (AOR 2.11; 95 % CI 1.21-4.50; p = 0.012). Smoking remained a strong predictor, with smokers nearly 2.76 times more likely to be pre-hypertensive than non-smokers (95 % CI 1.38-5.60; p = 0.006). A family history of hypertension doubled the odds (AOR 2.19; 95 % CI 1.30-3.50; p < 0.001), and a sedentary lifestyle as no weekly physical activity was associated with a 2.74-fold higher risk (95 % CI 1.05-6.80; p = 0.022). Sleeping less than six hours per day nearly doubled pre-hypertension odds (AOR 1.98; 95 % CI 1.10-3.20; p = 0.008). Notably, stress emerged as the strongest predictor, with those reporting stress having almost four times the odds of pre-hypertension (AOR 3.82; 95 % CI 2.37-6.10; p < 0.001.

**Table 4:** Multiple logistic regression analysis to find out the association between pre-hypertension and related variables “Enter method” was used for binomial logistic regression. * Statistically significant at 95% confidence interval. OR: odds ratio; AOR: adjusted odds ratio

S. No.	Variable	P value	AOR	95% CI
1.	Age above 22 years	0.034*	1.833	1.03 – 2.6
2.	Female gender	0.069	1.241	0.89 – 2.3
3.	Fourth year MBBS	0.012*	2.107	1.21 – 4.5
4.	Smoking habit	0.006*	2.755	1.38 – 5.6
5.	Family history of hypertension	<0.001*	2.186	1.3 – 3.5
6.	No physical activity /week	0.022*	2.738	1.05 – 6.8
7.	< 6 hours of sleep / day	0.008*	1.983	1.1 – 3.2
8.	Stress	<0.001*	3.822	2.37 – 6.1

## Discussion

Our study found that 28% of the 350 participants had pre-hypertension. This prevalence is notably higher than that reported in similar studies conducted among medical students across India. For example, research conducted in Andhra Pradesh  observed a pre-hypertension prevalence of 15.9%, with rates of 29.3% in men and 5.2% in women [20] while few other studies reported higher prevalence rates.A study from Mumbai identified pre-hypertension in 32.4% of medical students [21], while another research from Andhra Pradesh reported a prevalence of 37.45% [22]. Additionally, a separate study in Puducherry noted that 42.4% of medical students had elevated blood pressure [23]. The significant difference of higher prevalence in our study compared to these regions may indicate regional differences, potentially influenced by variations in lifestyle, stress levels, or genetic factors among medical students in Chengalpattu district.

As observed in this study, pre-hypertension was more prevalent among participants over the age of 22 years (46.9%) compared to their younger counterparts (53.1%), with a statistically significant association (p = 0.019). To support this observation our multiple logistic regression analysis revealed that students above the age of 22 were nearly twice as likely to develop pre-hypertension (AOR: 1.833; 95% CI: 1.03-2.6; p = 0.034). Similar trends have been documented in studies from Bahrain [24], Egypt [25], and Mumbai [21], all of which highlight age as a crucial risk factor for pre-hypertension and hypertension among university students. 

In our current study, female students exhibited a higher prevalence of pre-hypertension (63.3%) compared to male students (36.7%), with an odds ratio (OR) of 1.76 (95% CI: 1.08-2.86, p = 0.020). This contrasts with studies from Puducherry [23] and Bahrain [24], where male students demonstrated a higher prevalence of pre-hypertension (29.3% and 44.9%, respectively) compared to their female counterparts (5.2% and 17.7%, respectively). Similarly, a study in Mumbai identified male gender as a significant risk factor for hypertension among medical students [21]. In our study, the proportion of obesity and overweight was higher among female individuals than male individuals. This could have contributed to a higher proportion of pre-hypertensives among female individuals. The discrepancy between our findings and those from other studies suggests that cultural, environmental, or behavioral factors may influence blood pressure differently across populations.

In the present study, final-year medical students exhibited a notably higher prevalence of pre-hypertension (38.8%) compared to first-year students (15.3%), with a statistically significant association (OR = 2.17; 95% CI: 1.08-4.37; p = 0.030). Adding to this, multiple logistic regression analysis revealed that final-year students were more than twice as likely to develop pre-hypertension (AOR: 2.107; 95% CI: 1.21-4.5; p = 0.012), highlighting how the growing academic and clinical demands faced during the final years of medical education may contribute to increased cardiovascular risk. This finding aligns with a study from Andhra Pradesh, which reported an increasing prevalence of pre-hypertension with advancing academic years among medical students [22]. Similarly, research conducted in Bahrain [24] found higher rates of pre-hypertension among senior students compared to their junior counterparts, and a study in Mumbai [21]** **also observed a rise in hypertension prevalence as students progressed through academic years.

Students from nuclear families had a lower prevalence of pre-hypertension (54.1%) compared to those from joint families (45.9%), with an OR of 0.761 (95% CI: 0.47-1.22, p = 0.259). Limited studies have directly examined the association between family structure and pre-hypertension. However, family environment and associated stressors can influence blood pressure. A study in Egypt highlighted family history as a significant risk factor for hypertension among university students [25]. However, family dynamics and support systems may play a role in shaping stress levels and lifestyle habits, which in turn can influence blood pressure. Further investigation is required to explore this relationship in greater detail.

Smokers exhibited a significantly higher prevalence of prehypertension (26.5%) compared to non-smokers (73.5%), with an odds ratio of 2.492 (95% CI: 1.38-4.49, p = 0.002); participants with smoking habits were nearly three times more likely to develop pre-hypertension (AOR: 2.755; 95% CI: 1.38-5.6; p = 0.006), underscoring the harmful impact of tobacco use on cardiovascular health. Supporting this, a study in Puducherry by Kar et al. using the STEPS survey found that tobacco users had more than double the prevalence of prehypertension compared to non-users [6]. Research by Parthaje et al. in urban South India similarly reported that individuals who used tobacco (90.1%) had higher rates of prehypertension than non-users [26]. This collectively reinforces evidence from various Indian studies that underscore a strong association between smoking and elevated blood pressure, thereby highlighting the critical need for tobacco cessation programs as a key strategy to reduce the burden of hypertension in India.

No significant association was found between alcohol consumption and pre-hypertension, with an OR of 1.679 (95% CI: 0.88-3.20), p = 0.113, whereas a study done by Parthaje et al. identified alcohol as a significant risk factor for pre-hypertension and hypertension [26].

Participants with a family history of hypertension had a higher prevalence of pre-hypertension (62.2%) compared to those without (37.8%), with an OR of 1.809 (95% CI: 1.12-2.93), p = 0.015. Similarly, a study in Bahrain found a significant association between a family history of hypertension and elevated blood pressure among university students [24].

Obese participants had a higher prevalence of pre-hypertension (11.2%) compared to those with normal BMI (52%), with an OR of 1.39 (95% CI: 0.61-3.15), p = 0.073. A study in India reported a prevalence of overweight and obesity at 43%, with higher anthropometric indices associated with pre-hypertension. A study found that medical students had a 12.8% increased chance of having pre-hypertension with each unit increase in BMI [27]. A study demonstrated a strong positive correlation between BMI and blood pressure among medical students, suggesting weight control as a preventive measure against hypertension [28].

Although obese participants in our study showed a higher prevalence of pre-hypertension (11.2%) compared to those with a normal BMI (52%), with an OR of 1.39 (95% CI: 0.61-3.15; p = 0.073), the association was not statistically significant. In contrast, previous studies conducted in India by Bhavani et al. in Andhra Pradesh reported a combined prevalence of overweight and obesity at 43%, highlighting a significant link between elevated anthropometric indices and pre-hypertension [20]. One study done in Agartala [27] noted that medical students had a 12.8% increased likelihood of developing pre-hypertension with each unit increase in BMI, while another research from China demonstrated a strong positive correlation between BMI and blood pressure [28], emphasizing the importance of weight management as a preventive strategy. These findings underscore the need for continued efforts in promoting healthy lifestyles among young adults, even though our study did not observe a significant association.

Our study found that participants who exercised only once a week were significantly more likely to have pre-hypertension (OR: 3.67; p = 0.047) compared to those who were active more than five times a week. Even more strikingly, those who reported no physical activity at all during the week had nearly three times the odds of developing pre-hypertension (AOR: 2.738; 95% CI: 1.05-6.8; p = 0.022). These findings highlight just how important regular movement is for maintaining healthy blood pressure; even small, consistent efforts can make a meaningful difference. This aligns with evidence from a systematic review and meta-analysis, which showed that both aerobic and resistance training can help lower blood pressure in people with pre-hypertension [29]. Another study further emphasized the positive impact of combining physical activity with good sleep habits to support better blood pressure control [30]. Together, these insights remind us that staying active isn’t just about fitness-it’s a powerful step toward protecting long-term heart health.

In our study, students who slept less than six hours per day were found to have significantly higher odds of developing pre-hypertension (OR: 1.820; p = 0.024; AOR: 1.983; 95% CI: 1.1-3.2; p = 0.008), reflecting a growing body of evidence linking short sleep duration with increased blood pressure. Previous research has shown that sleeping fewer than seven hours per night is associated with a heightened risk of both pre-hypertension and hypertension, particularly among females [31]. Alongside sleep, stress also emerged as a major factor in our findings [32].

Students experiencing stress had over three times the odds of pre-hypertension (AOR: 3.822; 95% CI: 2.37-6.1; p < 0.001). A study done by Kar et al. shows similar results [6]. These results emphasize the urgent need to prioritize sleep hygiene, stress management, and mental health support within academic environments to help students maintain both their physical and emotional well-being.

Limitations of this study include the inability to generalize the findings to the entire young adult population of the Chengalpattu district, as the sample was limited to medical college students. Social desirability bias is common in this study. Dietary habits play a vital role in hypertension, which was not studied here. Furthermore, since most participants were having their blood pressure measured for the first time and readings were taken on a single day, there is a possibility of over-diagnosis, potentially influenced by white coat hypertension.

## Conclusions

Our study reveals a notably high prevalence of pre-hypertension among undergraduate medical students in the Chengalpattu district, emphasizing the early onset of cardiovascular risk factors in this young population. Key socio-demographic and behavioral variables were found to be significantly associated with elevated blood pressure levels. Students aged above 22 years exhibited a significantly higher risk of pre-hypertension, revealing the possible role of age-related stress, academic pressure, and lifestyle changes. Female students were more likely to be pre-hypertensive compared to their male counterparts, suggesting the need to explore gender-specific stressors and health behavior in this group. Final-year students had markedly increased odds of pre-hypertension compared to first-years, potentially due to cumulative academic stress and clinical responsibilities. Smoking emerged as a strong modifiable risk factor, with smokers having significantly higher odds of developing pre-hypertension than non-smokers. These findings underscore the importance of early identification and intervention, including regular blood pressure monitoring, lifestyle counseling, and targeted health education programs, especially for high-risk groups within the student community. Proactive measures can help mitigate long-term cardiovascular risks and foster a healthier future medical workforce.

## Appendices

Tool for Assessment of Prevalence of Pre-Hypertension and Associated Risk Factors Among Undergraduate Medical Students

Section A: Demographic Information

1.  Age:

2. Gender:

a) Male

b) Female

3. Year of study

a) 1st year

b) 2nd year

c) 3rd year

d) 4th year 

4. Type of family

a) Nuclear

b) Joint

5. Monthly income

6. Father’s educational status

a) Illiterate

b) Primary school

c) Secondary school

d) Graduate/diploma

e) Postgraduate

7. Father’s occupational status

a) Unemployed

b) Unskilled

c) Skilled

d) Semi-skilled

e) Professional

8. Mother’s educational status

a) Illiterate

b) Primary school

c) Secondary school

d) Graduate/diploma

e) Postgraduate

9. Mother’s occupational status

a) Unemployed

b) Unskilled

c) Skilled

d) Semi-skilled

e) Professional

10. Family history of hypertension?

a) Yes

b) No

11. History of drug abuse: Yes/No

a) Smoking: Yes/No

b) Alcohol: Yes/No

c) Any other: Yes/No, if yes ------------

Section B: Anthropometry

12. Height:

13. Weight:

14. BMI:

15. Blood pressure:

a) Systolic:                 Diastolic:

b) <120                       a) <80

c) 120-139                  b) 80-89

d) >140                       c) > 90

Section C: Academic, Psychosocial, and Environmental Determinants of Pre-Hypertension

16. How often do you engage in moderate to vigorous physical activity per week?

a) Less than 30 minutes

b) 30 minutes to 1 hour

c) 1 to 2 hours

d) More than 2 hours

17. Time spent sitting or being idle on a typical day?

a) > 6 hours

b) < 6 hours

18. Type of stay

a) Day scholar

b) Hosteller

19. Hours of sleep per day

a) < 6 hours

b) > 6 hours

20. Type of diet?

a) Mixed

b) Vegetarian

21. Suffering from any mental health conditions (e.g., anxiety, depression)?

a) Yes

b) No

22. How much time do you spend on social media and online platforms per day?

a) Hours/day.......

23. How often do you consume junk food in a typical week?

a) Never

b) 1-2 times

c) 3-4 times

d) 5 or more times

24. Are you stressed about anything? Yes/no

If Yes : Academic/Personal/ Financial/ Future/Self Image /Peer Pressure/ Others-------

Section D: Perceived Stress Scale [18]

Based on your experience in the last month circle your option for the following questions: 

0 - never  1 - almost never  2- sometimes  3 - fairly often  4 - very often 

How often have you been upset because of something that happened unexpectedly? 0

1

2

3

4

How often have you felt that you were unable to control the important things in your life?

0

1

2

3

4

How often have you felt nervous and stressed?

0

1

2

3

4

How often have you felt confident about your ability to handle your personal problems?

0

1

2

3

4

How often have you felt that things were going your way?

0

1

2

3

4

How often have you found that you could not cope with all the things that you had to do?

0

1

2

3

4

How often have you been able to control irritations in your life?

0

1

2

3

4

How often have you felt that you were on top of things?

0

1

2

3

4

How often have you been angered because of things that happened that were outside of your control?

0

1

2

3

4

How often have you felt difficulties were piling up so high that you could not overcome them?

0

1

2

3

4
